# Supplementary material for: Temperature stress induces mites to help their carrion beetle hosts by eliminating rival blowflies
Source: eLife. 2020 Aug 5;9:e55649. doi: 10.7554/eLife.55649 (PMC7431131; doi:10.7554/eLife.55649)
Supplement: Supplementary file 1. — (a) Results from the final models for the reproductive success of beetles, blowflies, and mites in the field experiment. The final models used were: glmer.nb(Number of larvae ~ Mite treatment*(poly(temperature,degree = 2)[,2]+ poly(temperature,degree = 2)[,1])+Carcass mass+(1|site)+(1|year)). Models analyzing burying beetle larvae and blowfly larvae were both sufficient to reject the null hypotheses, with 81.3% and 98.6% power, respectively, whereas the model analyzing mite offspring was not, with a power of 36.9%. (b) Results from the final models for the reproductive success of beetles, blowflies, and mites in the Laboratory Experiment 1. For beetles, the final model used was: glmer.nb(Number of larvae ~ Mite treatment*Temperature treatment*Blowfly treatment+Carcass mass+(1|block)); for blowflies, the final model used was: glmer.nb(Number of larvae ~ Mite treatment*Temperature treatment+Carcass mass+(1|block)); and for mites, the final model used was: glmer.nb(Number of larvae ~ Blowfly treatment*Temperature treatment+Mite treatment+Carcass mass+(1|block)). All these models were sufficient to reject the null hypotheses, with the 97%, 97%, and 98.2% power, for analyses of burying beetle larvae, blowfly larvae, and mite offspring, respectively. (c) Results from the final models for the development of blowfly larvae in the Laboratory Experiment 2. For number of blowfly larvae, the final model used was: glm.nb(Number of larvae ~ Temperature treatment+Carcass mass+Blowfly egg mass); for carcass consumption rate, the final model used was: betareg(Consumption rate ~Temperature treatment+Carcass mass+Blowfly egg mass); and for development rate, the final model used was: glmer(Days ~ Temperature treatment*Developmental stage+Carcass mass+Blowfly egg mass+(1|carcass ID)). Models analyzing number of blowfly larvae and carcass consumption rate were both not sufficient to reject the null hypotheses, with 12.9% and 22.8% power, respectively, whereas the model analyzing developm [file elife-55649-supp1.docx]

**Supplementary files**

| Supplementary File 1a. Results from the final models for the reproductive success of beetles, blowflies, and mites in the field experiment. The final models used were: glmer.nb(Number of larvae ~ Mite treatment*(poly(temperature,degree=2)[,2]+ poly(temperature,degree=2)[,1])+Carcass mass+(1\|site)+(1\|year)). Models analyzing burying beetle larvae and blowfly larvae were both sufficient to reject the null hypotheses, with 81.3% and 98.6% power, respectively, whereas the model analyzing mite offspring was not, with a power of 36.9%. | | | | |
| --- | --- | --- | --- | --- |
| **Dependent variables** | **Explanatory variables** | ***X^2^*** | **d.f.** | **p** |
| Number of beetle larvae | Mite treatment | 3.46 | 2 | 0.177 |
|  | Temperature | 0.25 | 1 | 0.616 |
|  | Temperature^2^ | **11.50** | **1** | **<0.001** |
|  | Carcass mass | 0.01 | 1 | 0.913 |
|  | Mite treatment * temperature | 0.67 | 2 | 0.716 |
|  | Mite treatment * temperature^2^ | **10.81** | **2** | **0.004** |
| Number of blowfly larvae | Mite treatment | **9.31** | **2** | **0.010** |
|  | Temperature | **5.09** | **1** | **0.024** |
|  | Temperature^2^ | **13.50** | **1** | **<0.001** |
|  | Carcass mass | **11.86** | **1** | **<0.001** |
|  | Mite treatment * temperature | 3.69 | 2 | 0.158 |
|  | Mite treatment * temperature^2^ | **11.53** | **2** | **0.003** |
| Number of mite offspring | Mite treatment | 0.08 | 1 | 0.778 |
|  | Temperature | 2.63 | 1 | 0.105 |
|  | Temperature^2^ | 1.07 | 1 | 0.300 |
|  | Carcass mass | 3.25 | 1 | 0.071 |
|  | Mite treatment * temperature | 0.02 | 1 | 0.877 |
|  | Mite treatment * temperature^2^ | 0.03 | 1 | 0.863 |
| p values < 0.05 are statistically significant, in bold | |  |  |  |

| Supplementary File 1b. Results from the final models for the reproductive success of beetles, blowflies, and mites in the Laboratory experiment 1. For beetles, the final model used was: glmer.nb(Number of larvae ~ Mite treatment*Temperature treatment*Blowfly treatment+Carcass mass+(1\|block)); for blowflies, the final model used was: glmer.nb(Number of larvae ~ Mite treatment*Temperature treatment+Carcass mass+(1\|block)); and for mites, the final model used was: glmer.nb(Number of larvae ~ Blowfly treatment*Temperature treatment+Mite treatment+Carcass mass+(1\|block)). All these models were sufficient to reject the null hypotheses, with the 97%, 97%, and 98.2% power, for analyses of burying beetle larvae, blowfly larvae, and mite offspring, respectively. | | | | |
| --- | --- | --- | --- | --- |
| **Dependent variables** | **Explanatory variables** | ***X^2^*** | **d.f.** | **p** |
| Number of beetle larvae | |  |  |  |
| *Full model* | Mite treatment | 2.46 | 2 | 0.293 |
|  | Temperature treatment | 2.66 | 2 | 0.265 |
|  | Blowfly treatment | **27.22** | **1** | **<0.001** |
|  | Carcass mass | **10.02** | **1** | **0.002** |
|  | Mite treatment * temperature treatment | 1.54 | 4 | 0.819 |
|  | Mite treatment * blowfly treatment | **10.27** | **2** | **0.006** |
|  | Temperature treatment * blowfly treatment | **15.54** | **2** | **<0.001** |
|  | Mite treatment * temperature treatment * blowfly treatment | **10.33** | **4** | **0.035** |
| *Without blowflies* | Mite treatment | **10.60** | **2** | **0.005** |
|  | Temperature treatment | 4.09 | 2 | 0.129 |
|  | Carcass mass | **15.43** | **1** | **<0.001** |
| *With blowflies* | Mite treatment | 5.85 | 2 | 0.054 |
|  | Temperature treatment | **18.73** | **2** | **<0.001** |
|  | Carcass mass | 2.36 | 1 | 0.125 |
|  | Mite treatment * temperature treatment | **11.39** | **4** | **0.023** |
| Number of blowfly larvae | Mite treatment | **8.83** | **2** | **0.012** |
|  | Temperature treatment | **34.05** | **2** | **<0.001** |
|  | Carcass mass | **4.36** | **1** | **0.037** |
|  | Mite treatment * temperature treatment | **14.33** | **4** | **0.006** |
| Number of mite offspring | Mite treatment | **12.60** | **1** | **<0.001** |
|  | Temperature treatment | **10.12** | **2** | **0.006** |
|  | Blowfly treatment | **10.85** | **1** | **<0.001** |
|  | Carcass mass | **4.41** | **1** | **0.036** |
|  | Blowfly treatment * temperature treatment | 5.58 | 2 | 0.061 |
| p values < 0.05 are statistically significant, in bold | |  |  |  |

| Supplementary File 1c. Results from the final models for the development of blowfly larvae in the Laboratory experiment 2. For number of blowfly larvae, the final model used was: glm.nb(Number of larvae ~ Temperature treatment+Carcass mass+Blowfly egg mass); for carcass consumption rate, the final model used was: betareg(Consumption rate ~ Temperature treatment+Carcass mass+Blowfly egg mass); and for development rate, the final model used was: glmer(Days ~ Temperature treatment*Developmental stage+Carcass mass+Blowfly egg mass+(1\|carcass ID)). Models analyzing number of blowfly larvae and carcass consumption rate were both not sufficient to reject the null hypotheses, with 12.9% and 22.8% power, respectively, whereas the model analyzing development rate of blowfly larvae was highly sufficient, with a power of 100%. | | | | |
| --- | --- | --- | --- | --- |
| **Dependent variables** | **Explanatory variables** | ***X^2^*** | **d.f.** | **p** |
| Number of blowfly larvae | Temperature treatment | 0.35 | 2 | 0.841 |
|  | Carcass mass | 0.64 | 1 | 0.423 |
|  | Blowfly egg mass | 1.25 | 1 | 0.263 |
| Carcass consumption rate | Temperature treatment | 2.57 | 2 | 0.277 |
|  | Carcass mass | 3.08 | 1 | 0.079 |
|  | Blowfly egg mass | 0.33 | 1 | 0.564 |
| Development rate of blowfly larvae | Temperature treatment | **19.06** | **2** | **<0.001** |
|  | Developmental stage | **405.39** | **4** | **<0.001** |
|  | Carcass mass | 0.12 | 1 | 0.731 |
|  | Blowfly egg mass | 3.25 | 1 | 0.072 |
|  | Temperature treatment * Developmental stage | **178.46** | **8** | **<0.001** |
| p values < 0.05 are statistically significant, in bold | |  |  |  |

| Supplementary File 1d. Results from the final models for beetle's carcass preparation in the Laboratory experiment 3. For number of blowfly larvae, the final model used was: glm.nb(Number of larvae ~ Temperature treatment+Carcass mass+Blowfly egg mass); and for carcass roundness, the final model used was: glm.nb(Roundness ~ Temperature treatment+Carcass mass+Blowfly egg mass). Models analyzing number of blowfly larvae and carcass roundness were both sufficient to reject the null hypotheses, with 96.4% and 99.5% power, respectively. | | | | |
| --- | --- | --- | --- | --- |
| **Dependent variables** | **Explanatory variables** | ***X^2^*** | **d.f.** | **p** |
| Number of blowfly larvae | Temperature treatment | **14.08** | **2** | **<0.001** |
|  | Carcass mass | 0.42 | 1 | 0.516 |
|  | Blowfly egg mass | 3.77 | 1 | 0.052 |
| Carcass roundness | Temperature treatment | **30.30** | **2** | **<0.001** |
|  | Carcass mass | 2.16 | 1 | 0.142 |
|  | Blowfly egg mass | 2.61 | 1 | 0.106 |
| p values < 0.05 are statistically significant, in bold | |  |  |  |
